# Community‐Driven Research Priorities for Genetic Counseling: Shaping the Future of Genetic Counseling Research by Centering Multicultural Perspectives

**DOI:** 10.1002/jgc4.70270

**Published:** 2026-07-28

**Authors:** Victoria R. Johnson, Elena R. Fisher, Kathleen A. Culhane‐Pera, Crystal Y. Lumpkins, Abdirashid Shire, Mariana Ramírez, Aida Parra de Young, Carolina E. Uribe Abad, Eric Yang, Sey Lee, Abdiziz Abdi, J. Michelle Vann, Stephanie Moss, Heather A. Zierhut

**Affiliations:** ^1^ Department of Genetics, Cell Biology, and Development University of Minnesota Minneapolis Minnesota USA; ^2^ SoLaHmo Partnership for Health and Wellness Community‐University Health Care Center Minneapolis Minnesota USA; ^3^ Huntsman Cancer Institute Salt Lake City Utah USA; ^4^ Department of Communication The University of Utah Salt Lake City Utah USA; ^5^ Shire Scientific, LLC Minneapolis Minnesota USA; ^6^ Department of Population Health, JUNTOS Center for Advancing Latino Health University of Kansas Medical Center Kansas City Kansas USA; ^7^ Community Advisory Board JUNTOS Center for Advancing Latino Health Kansas City Kansas USA; ^8^ Community Advisory Board Kansas University Cancer Center Masonic Cancer Alliance Kansas City Kansas USA; ^9^ Hmong GC‐PRO Community Advisory Board Minneapolis Minnesota USA; ^10^ Shire Somali GC‐PRO Community Advisory Board Minneapolis Minnesota USA; ^11^ Faith Works “Connecting for a Healthier Community” Community Advisory Board Kansas City Kansas USA

**Keywords:** card sort, community partnerships, community‐based participatory action research, community‐based participatory research, future, genetic counseling, genetics education, genomics, research priorities, research recommendations

## Abstract

Community‐based participatory research (CBPR), a research approach that centers the collective strengths of community‐researcher partnerships, can direct future genetic counseling (GC) research initiatives toward issues that are critical to communities most impacted by genomic healthcare disparities. Co‐developed, innovative solutions to persistent research gaps may help address community access to and education about genomic healthcare. This study utilized a CBPR approach to develop a prioritized list of GC research directions that align with the goals and needs of representatives from racially/ethnically underrepresented communities in research. Twelve members from four community advisory boards (CABs) representing the Hispanic/Latino, Black/African American, Hmong, and Somali communities came together to complete three card sorting activities. Twenty cards describing research project ideas were developed through inductive qualitative content analysis of 22 meetings held with individual CABs (April 2023 to May 2025). Three members from each CAB first sorted their own CAB's project idea cards. Then, members were divided into three new small groups with one member from each CAB. Cross‐CAB groups completed a second card sorting activity to identify their most important project ideas, followed by a third sorting activity to prioritize their top card. The cross‐CAB identified three research priorities: (A) work with primary care clinics to encourage more people to see a genetic counselor, (B) learn which GC techniques are best according to patients and the community, and (C) investigate whether educational projects make people more aware of GC or not. By centering multicultural perspectives in the creation of a GC research agenda, the future of GC research can be better aligned with the priorities of communities and eventually support novel, tailored strategies to address genomic healthcare disparities.

## Abstract (highlights)

**What Is Known About This Topic:**

- Genetic counseling (GC) research priorities are generally determined by funding agencies, scientific review panels, and promoted by professional organizations.
- The needs, values, and goals of patients and communities should be prioritized in GC research initiatives in order to identify the most important research topics for which innovative solutions should be developed.

**What This Paper Adds to the Topic:**

- A community‐driven prioritized GC research agenda was created through a series of card sorting activities with representatives from four multicultural community advisory boards.
- This is the first time, to our knowledge, that prioritized research directions have been co‐developed and confirmed by community members, which include: (A) work with primary care clinics to encourage more people to see a genetic counselor, (B) learn which GC techniques are best according to patients and the community, and (C) investigate whether educational projects make people more aware of GC or not.

## 1 Introduction

Genetic counseling (GC) has demonstrable value in both patient health outcomes and medical system efficacy and, as a result, this service has rapidly become an essential component of both preventive and diagnostic healthcare (Cook et al. 2025; Culver et al. 2024; Schienda and Stopfer 2020). With the expansion of GC, disparities in who accesses and benefits from genetics services continue to broaden as well, with non‐Hispanic White, educated, privately insured individuals being more likely to receive genetic services (Armstrong et al. 2005; Bellaiche et al. 2021; Cragun et al. 2017; Lin et al. 2021; McCarthy et al. 2016; Reid et al. 2020; Southwick et al. 2020; Wagner et al. 2024; Wideroff et al. 2004; Yedjou et al. 2019). Research addressing the root causes of health disparities in GC as well as effective methods to mitigate these disparities has been more recently emphasized as one of the top research priorities for the field by the National Society of Genetic Counselors (Senter et al. 2020). However, the individuals and target populations most impacted by disparities in genomic healthcare are most often not consulted in the design of such research endeavors (Zierhut et al. 2024).

In GC research specifically, a scoping literature review recently found that community members are less frequently involved in the development of research questions and in the creation of research products or interventions (Zierhut et al. 2024). These gaps in research involvement may cause community goals to be excluded, misinterpreted, or not prioritized. Correspondingly, interventions intended to address GC health disparities may not be tailored to the target population's values or cultural norms, or may completely disregard the community's needs and preferences altogether (Concannon et al. 2014; Zayhowski et al. 2025). The divergence between researcher‐led initiatives and community‐centered research highlights a pressing need to intentionally collaborate with members of groups underrepresented in research, such as racial and ethnic communities, in order to determine the most appropriate and effective ways to address health disparities in genomics care. Leveraging the expertise of community‐researcher partnerships can support more innovative and successful research interventions, which in turn are more likely to be accepted and adopted by patients and integrated into health systems long‐term to bring benefits back to the community.

Community‐based participatory research (CBPR) is a research approach that intentionally involves members of specific communities (often racially and ethnically diverse or historically underrepresented groups) in research initiatives by building genuine, co‐led, and equitable relationships with researchers (Israel et al. 2010; Maiter et al. 2008; Wallerstein et al. 2017; Zayhowski et al. 2025). CBPR is considered a restorative justice approach to research that centers the collective strengths of community‐researcher partnerships to ultimately improve community health (Israel et al. 1998). The timing and extent of community involvement in CPBR exists along a continuum, and different involvement strategies influence the outcome of these projects (Key et al. 2019). CBPR is grounded in certain gold standard principles, including that the research (1) recognizes the community as a unit of identity, (2) builds upon the strengths and resources of the community, (3) facilitates collaborative partnerships in all phases of the research, (4) integrates knowledge and action for mutual benefit of all partners, (5) promotes a co‐learning and empowering process that attends to social inequalities, (6) involves a cyclical and iterative process, (7) addresses health from both positive and ecological perspectives, (8) disseminates findings and knowledge gained to all partners, and (9) involves a long‐term process and commitment to sustainability (Israel et al. 1998, 2010). Communities should be involved throughout the research process, including from formation of the research question to dissemination of results, in order to create the greatest impact (Israel et al. 1998, 2010; Wallerstein et al. 2017; Zierhut et al. 2024). Community‐based participatory action research (CBPAR or CPAR) emphasizes one CBPR principle, focusing on taking action based on research findings to create positive social change within communities (Maiter et al. 2008; McTaggart 1997).

Though CBPR approaches have only been reported in GC research settings within the past two decades, these practices have already demonstrated success both in exploratory projects focused on understanding community perspectives as well as in intervention‐based GC research (Zierhut et al. 2024). Exploratory CBPR projects may glean community opinions on GC services through interviews, focus groups, and workshops. Through these methods, researchers can better understand culture‐specific phenomena and highlight barriers to genetics services that community members themselves have faced. This information may then be utilized to guide research toward initiatives that align with community goals (Cheung et al. 2019; Holzer et al. 2021; Horowitz et al. 2017; Lumpkins et al. 2020, 2023). Interventional CBPR projects have allowed for co‐creation of products appropriate for a specific community's needs, such as culturally‐tailored family history toolkits, educational leaflets about reproductive decisions, and booklets explaining what to expect in a GC appointment (Ali et al. 2019; Li et al. 2020; Linares and Ramirez 2023; Permuth‐Wey et al. 2010). When community needs and expertise are centered in the development of these products, community members demonstrate better understanding of genetic information as well as increased adherence to medical recommendations (Glanz et al. 2007; Hurtado‐de‐Mendoza et al. 2019; Jibaja‐Weiss et al. 2011; Shete et al. 2023; Sussner et al. 2010).

The present study utilized a collaborative, exploratory approach grounded in CBPR gold standard principles to develop a prioritized list of GC research directions that align with the goals and needs of representatives from racially and ethnically underrepresented communities (Israel et al. 1998, 2010). Specifically, the study's overarching goal was to bring together members of four community advisory boards CABs representing the Hispanic/Latino, Black/African American, Hmong, and Somali communities to collectively determine the most crucial areas of GC research. Our aim was to prioritize a research agenda for future GC research using card sorting activities. By centering multicultural perspectives and values in the creation of a prioritized GC research agenda, the future of GC research has the potential to be transformed from top‐down researcher‐led efforts to community‐driven research ideas that, when CBPR principles are integrated, can lead to high‐impact and transformational outcomes for communities.

## 2 Methods

This project was submitted to the University of Minnesota Institutional Review Board (IRB) in May 2025. While the project (STUDY00025540) was exempt from review, the authors followed standard ethical research standards. All CAB members were informed at the start of the meeting about the purpose of the project, extent of study participation, confidentiality considerations, and option to withdraw from participation at any time.

### 2.1 Origin and Make‐Up of Individual CABs

Four community‐specific GC CABs were formed in 2023 for the primary purpose of guiding the national, multi‐site Genetic Counseling Processes Result in Outcomes (GC‐PRO) study which is focused on improving the quality and efficiency of GC health communication in diverse populations (Fisher et al. 2024). All CABs were formed through longstanding research relationships with the respective CAB leaders and one author (H.A.Z.), who has experience working with CABs in other projects (Cheung et al. 2019; Holzer et al. 2021).

Two CABs were pre‐existing groups with existing organizational resources and support. Specifically, the JUNTOS Latina CAB is a pre‐existing group from the University of Kansas Medical Center “JUNTOS Center for Advancing Latino Health.” The JUNTOS CAB (led by M.R.) is composed of five Hispanic/Latina women who are involved in a variety of community health‐related work and who have varying experiences with GC, cancer, and genetic testing. The faith‐based Black/African American CAB is also a pre‐existing CAB from the “Faith Works: Connecting for a Healthier Community” organization based out of Kansas City, MO. The Faith Works CAB (led by C.L.) consists of eight men and women who primarily have experience with hereditary cancer and genetic testing.

Two CABs were developed as part of the GC‐PRO study: the Shire Somali CAB and the Hmong CAB. The Shire Somali CAB (led by A.S.) is composed of eight Somali men and women with a broad range of backgrounds, from pediatricians to parents to accountants. The Hmong CAB (led by K.A.C.‐P.) is composed of six Hmong community members who have in‐depth healthcare expertise as well as personal experience with genetic conditions. Both the Somali and Hmong CABs are local to Minnesota, where these immigrant communities are prevalent (Coritz et al. 2023; Monte and Shin 2022).

As part of the GC‐PRO study, each CAB met individually with the research team (H.A.Z. and E.R.F.) two to three times per year. The research team attempted to create an environment of shared power through recognition of the unique expertise of community members as well as meaningful inclusion of CAB members in decision‐making about study directives (Israel et al. 1998, 2010). The planned research‐related content of each meeting was consistent across the 4 CABs and included GC‐PRO study progress as well as various topics including access to GC, research participation of minoritized groups, and GC health communication strategies, amongst others. Discussions regarding CAB‐specific goals and interests also occurred, leading to organic divergence in the content of meetings. Research‐related content materials (e.g., proposed agendas) were sent via email prior to the meetings and reviewed with CAB leaders before the start of the meetings based upon their preferences. Each meeting involved co‐learning and capacity‐building surrounding GC and community needs as well as iterative discussions about the study progress and findings (Burke et al. 2013; Israel et al. 1998; Maiter et al. 2008). A total of 22 meetings were held with the individual CABs from April 2023 to May 2025 (JUNTOS: 6 meetings, hybrid; Faith Works: 6 meetings, virtual; Shire Somali: 5 meetings, in person; Hmong: 5 meetings, virtual). Detailed notes about meeting content and discussions were taken (by E.R.F.) for each of the 22 CAB meetings and distributed to the CABs after each meeting for consensus.

### 2.2 Formation of the Cross‐CAB

During individual CAB meetings, members from two CABs (e.g., Faith Works and JUNTOS CABs) expressed their interest in bringing together all four CABs to understand similarities and differences in each group's perspectives about GC research. In pursuit of this endeavor and with all 4 CABs' permission, authors E.R.F. and H.A.Z. wrote a grant proposal based upon the CABs' ideas which was subsequently funded through the University of Minnesota Office for Discovery and Translation in October 2024.

Following funding of the project, authors E.R.F. and H.A.Z. met with each individual CAB to identify two to three representatives from each CAB to participate in the in‐person cross‐CAB meeting. The research team first communicated logistics of participation and associated incentives (e.g., travel, lodging, and meals compensated, as well as a $400 honorarium per CAB member for engaging in the research meeting) with each CAB. Then, CABs independently identified representatives to join the cross‐CAB. The process for representative selection varied by CAB, according to CAB members' and CAB leaders' preferences. For example, one CAB underwent a democratic voting process to identify their CAB representatives based on CAB members' expressed interest and experience relevant to the project. Other CABs nominated specific CAB members or simply asked for volunteers who were willing and available to contribute to the project. Ultimately, two members and the leader from each individual CAB (e.g., JUNTOS, Faith Works, Shire Somali, and Hmong CABs) were confirmed to form the cross‐CAB (*n* = 12 members total). The 12 cross‐CAB members have a broad spectrum of personal, professional, and cultural backgrounds, and many have professional degrees and roles within health and wellness spaces. Collectively, the cross‐CAB represents trusted community leaders who are intentional about advocating for approaches that reflect the full range of community experiences. Communication to all members of the original CABs occurred to ensure agreement and transparency regarding the final cross‐CAB make‐up.

### 2.3 Content Analysis of CAB Meeting Notes

To address the goal of identifying prioritized recommendations for GC research, authors V.R.J. and H.A.Z. conducted an inductive qualitative content analysis (commonly referred to as conventional content analysis) using a pragmatic paradigm to identify research priorities and/or community goals that had already been shared by CAB members (Erlingsson and Brysiewicz 2017; Hsieh and Shannon 2005; Lynch et al. 2024). The authors conducted the following: (1) collected meeting notes for each CAB meeting, (2) initial read and review of notes by authors V.R.J. and H.A.Z., (3) discussion and creation of a list of preliminary codes, (4) individual coding by author V.R.J., (5) review of codes by H.A.Z., (6) inductive creation of categories, and (7) re‐review of all meeting notes and auditing of initial codes for reliability.

All of the notes from the 22 CAB meetings held from April 2023 to May 2025 were included in the analysis. Author V.R.J. read all CAB meeting notes to achieve immersion and holistic appreciation of the data. She then created preliminary codes to describe topics, opinions, and/or ideas raised by CAB members that were related to GC, genetic testing, and/or genetics‐related research. Topics raised by the research team and/or topics not related to genetics were not coded because the focus of the present study was on community research interests related to genetics. H.A.Z., who was present for all CAB meetings, reviewed meeting notes and preliminary topic coding. Three categories were created using the inductive approach to separate topics of discussions: research interests (e.g., research directions, considerations for conducting research, etc.); education‐focused topics (e.g., spreading awareness of GC, how to educate community members about GC and genetics concepts, etc.); and clinical‐related issues (e.g., barriers and facilitators to GC access, recommendations for clinicians, etc.). H.A.Z. and V.R.J. then sorted all codes into the three categories. V.R.J. subsequently reviewed all CAB meeting notes again, re‐coded codes as appropriate, and confirmed categorization before final review. Lastly, H.A.Z. reviewed the final coding and discussed any areas of ambiguity with V.R.J. to achieve concordance.

### 2.4 Creation of Project Idea Cards

Following the content analysis, education‐focused and clinical‐related codes were eliminated due to the focus on research topics of interest. Within the research interests category, codes that referenced research initiatives that were already in progress by the research team were excluded as the focus of the present study was to prioritize unexplored research topics. Codes that referenced strategies for conducting research within minoritized communities were also excluded because these recommendations focused on how to do the research rather than on specific research project topic ideas.

V.R.J. translated the remaining codes to phrases which would later be printed on project idea cards for the card sorting activities (see Section 2.5). V.R.J. attempted to clarify the language of these codes without modifying the underlying meaning or focus of the project ideas, in order to present the concept of the original code in understandable language without the use of research jargon. This process resulted in four distinct lists of potential topics for future research projects that reflected interests and priorities unique to each CAB. Notably, there was overlap of topics among the four CABs. Where possible, topic language was made consistent across CABs as long as it still accurately described the original code from each specific CAB.

From the four unique lists of potential research topics, V.R.J. and H.A.Z. used an iterative process to identify a total of five project idea cards per CAB to be used in the cross‐CAB card sorting activities. Five project ideas were chosen per CAB to ensure feasibility of completing the card sorting activities in the time allotted. Criteria used to define the final CAB‐specific card lists included: (1) topics that arose multiple times within a CAB; (2) topics that arose across multiple CABs; and (3) project ideas that could address multiple goals or interests expressed by CAB members. H.A.Z. independently audited the final list of topics. Ultimately, four sets of five project idea cards (e.g., five cards per CAB) were created reflecting both distinct ideas from each CAB as well as overlap in interests amongst all four CABs.

### 2.5 Cross‐CAB Meeting and Card Sorting Activities

The cross‐CAB meeting occurred on August 9, 2025 at the University of Minnesota‐Twin Cities campus. Cross‐CAB members were invited to an introductory dinner the day before the meeting to share their goals for cross‐CAB participation as well as any information about their cultural background(s) that might be helpful for others to know during the meeting the following day.

**FIGURE 1:**
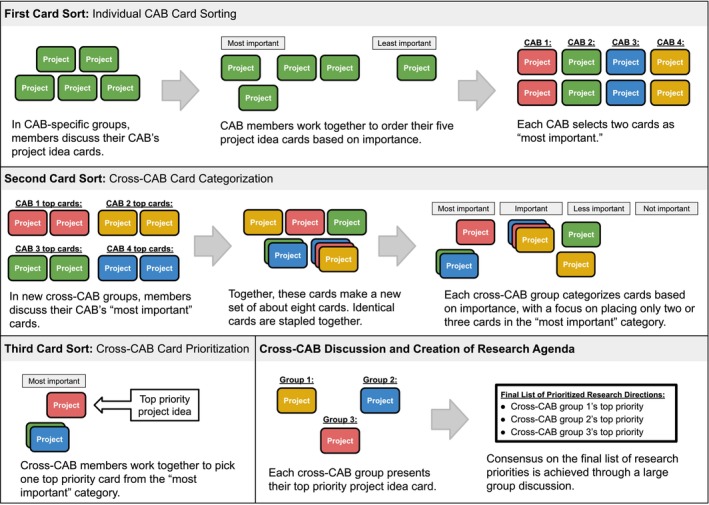
Series of card sorting activities used throughout the cross‐CAB meeting. An example of an individual CAB card sort is shown, where CABs ordered a small set of project idea cards based on importance. Cross‐CAB groups then completed the second card sort to categorize a larger set of project idea cards based on importance, ultimately selecting their top priority project to create the final list of research directions.

At the start of the meeting, H.A.Z. emphasized the goals of the meeting, specifically that this was an opportunity to work toward a shared aspiration of identifying prioritized GC research goals and/or interests of multiple communities that would have the widest reach and benefit. Author V.R.J. introduced the general procedure for the meeting, including that cross‐CAB members would be completing card sorting activities within small groups and contributing to small and large group discussions about the activities (Figure 1). Cross‐CAB members were encouraged to reach consensus within their small groups when making decisions during each card sorting activity before moving onto the next activity.

#### 2.5.1 First Card Sort: Individual CAB Card Sorting

The goal of the first card sorting activity was to order CAB‐specific project idea cards on a 5‐point Likert scale from most important to least important. Cross‐CAB members were assigned at tables to create four small groups of two members and one leader from each individual CAB. Cross‐CAB members were each provided with their own copy of their CAB's unique set of potential research topic cards. V.R.J. informed cross‐CAB members how the potential research topic cards were created (e.g., review of prior CAB notes) and encouraged members to discuss how the ideas arose at prior CAB meetings. V.R.J. then instructed members to collaboratively order the five cards from most important to least important, with particular emphasis on eventually determining the top two most important cards for their group.

One student note‐taker (undergraduate or graduate students involved in genetic counseling research) and one M.S. graduate student or UMN faculty member facilitator (e.g., principal or co‐investigators on the GC‐PRO study) were assigned to each table. Note‐takers audio recorded each activity and took written notes about the group discussions. Facilitators were instructed to evoke personal experiences and opinions about the cards and facilitate decision‐making when ranking the cards.

#### 2.5.2 Second Card Sort: Cross‐CAB Card Categorization

After the first activity, cross‐CAB members were randomly reassigned to new small groups made up of one member from each of the four CABs. CAB leaders were intentionally placed in the same small group given their similar levels of research expertise and to balance power dynamics among the other groups. V.R.J. invited cross‐CAB members to share their CAB's two most important cards from the previous activity and explain why each of the cards was selected by their CAB. Each member's two most important cards were then added to a pile to create a new set of eight project idea cards for each group.

As project idea cards overlapped amongst CABs, there was the possibility that some CABs may have selected the same card as most important. If two or more CABs selected the same project idea card, the cards were stapled together and treated as a single project idea card. Additionally, groups were allowed to “rescue” a card(s) that were not selected in the prior activity if members strongly wanted to consider it in the present activity.

V.R.J. instructed cross‐CAB members to collaboratively assign each potential research topic card to one of four importance categories: most important, important, less important, and not important. Emphasis was placed on only including two or three cards in the “most important” category. Each group was again joined by one note‐taker and one facilitator who performed the same prior responsibilities.

#### 2.5.3 Third Card Sort: Cross‐CAB Card Prioritization

For the third card sorting activity, cross‐CAB members remained in the same groups from the previous activity (e.g., one representative from each CAB in each small group). Using only the project idea cards determined to be in the “most important” category during the previous activity, V.R.J. instructed cross‐CAB members to determine which project idea card is the top priority for future research to address. To help contextualize this decision, cross‐CAB members were instructed to think of the top most crucial issue or idea related to GC research that all researchers should pursue from their community perspectives.

Facilitators encouraged members to only select one project idea card for this activity but reminded them that excluding a card does not negate its importance. Note‐takers recorded which project idea card was selected as the top priority.

#### 2.5.4 Cross‐CAB Discussion and Creation of Research Agenda

Lastly, V.R.J. invited cross‐CAB members to share which project idea card their group selected as the top priority. Members were encouraged to share additional context behind their group's thought process and reasoning for selecting their project idea card, including any cultural or community considerations that contributed to their decision. The meeting concluded with a large group discussion led by V.R.J. and H.A.Z. to elicit cross‐CAB members' opinions on the final list, including essential considerations for researchers who may choose to pursue these project ideas.

## 3 Results

### 3.1 Creation of CAB Project Idea Cards Through Content Analysis of Individual CAB Notes

The content analysis of individual CAB meeting notes yielded a total of 20 cards (e.g., four sets of five cards per CAB), 11 of which described unique project ideas (Table S1). Project ideas spanned broad research topic categories including: work with primary care clinics, GC techniques, community education, challenges to conducting research, epidemiology, and GC patient demographics.

### 3.2 First Card Sort: Individual CAB Card Sorting

**FIGURE 2:**
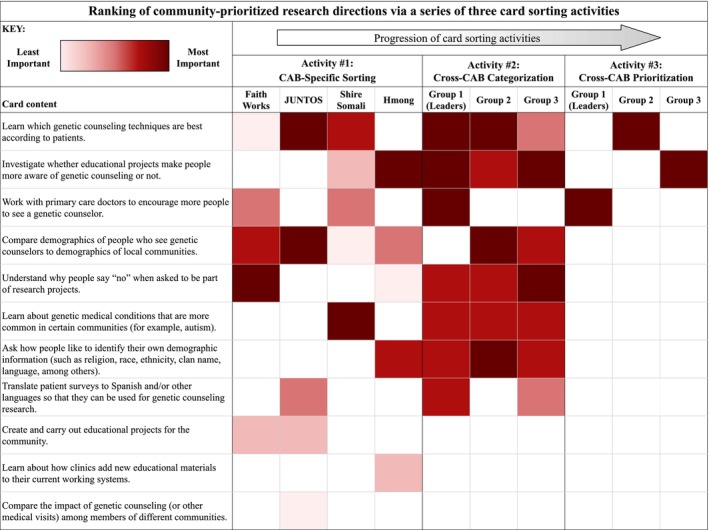
Ranking of community‐prioritized research directions via a series of three card sorting activities. Categorization of all project idea cards is shown through a series of three card sorting activities (left to right orientation representing progression through activities). Cards were sorted from least to most important, as indicated via heat map colorization gradient. Cards deemed most important across the three activities are highlighted at the top of the figure, with cards receiving lower importance scores ordered toward the bottom (top to bottom orientation representing CAB‐ordered importance).

For the first card sorting activity, CAB members sorted their unique sets of project idea cards using a five‐point scale from most important to least important (Figure 2, “Activity #1”). In general, there was some but not a lot of overlap within each group.

The first and second most important project idea cards selected by Faith Works CAB members were: (1) understand why people say “no” when asked to be part of research projects, and (2) compare demographics of people who see genetic counselors to demographics of local communities.

Due to time constraints, JUNTOS CAB members did not specifically order the first and second most important project idea cards. In no particular order, the two most important cards were: (A) compare demographics of people who see genetic counselors to demographics of local communities, and (B) learn which GC techniques are best according to patients. The next most important card was: translate patient surveys to Spanish and/or other languages so that they can be used for GC research.

The first and second most important project idea cards selected by Shire Somali CAB members were: (1) learn about genetic medical conditions that are more common in certain communities (e.g., autism), and (2) learn which GC techniques are best according to patients.

The first and second most important project idea cards selected by Hmong CAB members were: (1) investigate whether educational projects make people more aware of GC or not, and (2) ask how people like to identify their own demographic information (such as religion, race, ethnicity, clan name, and language, amongst others).

### 3.3 Second Card Sort: Cross‐CAB Card Categorization

For the second card sorting activity, cross‐CAB groups categorized their project idea cards based on their importance on a four‐point scale (e.g., most important, important, less important, not important). After rescuing cards, eliminating cards, and/or condensing multiple cards describing the same project idea into a single card, each group was left with six to seven unique cards for this step. The results for each group are presented in Figure 2 (“Activity #2”). The majority of cards amongst groups were placed into the “most important” and “important” categories. Only one group placed cards in the “less important” category, and zero groups placed cards in the “not important” category.

The first cross‐CAB group was made up of leaders from each of the four CABs. Members of this group rescued two cards and eliminated one card for this activity. Therefore, seven project idea cards were categorized. Three cards were placed in the “most important” category, and four cards were placed in the “important” category. In no particular order, the three cards in the “most important” category were: (A) investigate whether educational projects make people more aware of GC or not, (B) work with primary care doctors to encourage more people to see a genetic counselor, and (C) learn which GC techniques are best according to patients. The two rescued project idea cards were: (A) work with primary care doctors to encourage more people to see a genetic counselor, and (B) translate patient surveys to Spanish and/or other languages so that they can be used for GC research. The eliminated project idea card was: compare demographics of people who see genetic counselors to demographics of local communities.

The second cross‐CAB group categorized six project idea cards, none of which were rescued or eliminated. Three cards were placed in the “most important” category, and three cards were placed in the “important” category. In no particular order, the three cards in the “most important” category were: (A) learn which GC techniques are best according to patients, (B) compare demographics of people who see genetic counselors to demographics of local communities, and (C) ask how people like to identify their own demographic information (such as religion, race, ethnicity, clan name, and language, amongst others).

The third cross‐CAB group rescued one project idea card and therefore categorized seven total cards. Two cards were placed in the “most important” category, three cards were placed in the “important” category, and two cards were placed in the “less important” category. In no particular order, the two cards in the “most important” category were: (A) understand why people say “no” when asked to be part of research projects, and (B) investigate whether educational projects make people more aware of GC or not. The rescued project idea card was: translate patient surveys to Spanish and/or other languages so that they can be used for GC research.

### 3.4 Third Card Sort: Cross‐CAB Card Prioritization

For the third card sorting activity, each cross‐CAB group reached consensus in their prioritization of a single project idea card. The results for each group are presented in Figure 2 (“Activity #3”). Notably, all three groups chose a different top priority topic.

The first cross‐CAB group, consisting of leaders from each CAB, chose the following project idea card as the highest priority: work with primary care doctors to encourage more people to see a genetic counselor. The second cross‐CAB group chose the following project idea card as the highest priority: learn which GC techniques are best according to patients. The third cross‐CAB group chose the following project idea card as the highest priority: investigate whether educational projects make people more aware of GC or not.

### 3.5 Cross‐CAB Large Group Discussion and Creation of Research Agenda

Each cross‐CAB group presented their top priority project idea card to the large group and discussed their reasoning for selecting the card, including any recommendations to clarify the purpose of or strategies to address the project. Together, these cards created the finalized research agenda consisting of topics that community members have identified as top priorities (Figure 2): (A) work with primary care clinics to encourage more people to see a genetic counselor, (B) learn which GC techniques are best according to patients and the community, and (C) investigate whether educational projects make people more aware of GC or not.

Cross‐CAB members expressed that many project idea cards were inherently connected to one another, such that a project addressing one issue indirectly addresses or prompts another. In discussing their challenges deciding which projects to ultimately prioritize, members noted that a strategic approach is necessary in order to maximize limited available resources to develop effective research interventions that reach and benefit a variety of patient communities, all of which have individual needs and goals. The cross‐CAB's strategy is illustrated by the final list of research priorities, which notably is not wholly representative of all individual CAB‐specific priorities identified in the first card sorting activity. Rather, the final list reflects cross‐CAB members' aim to pursue research initiatives that maximize the benefits for a greater number of communities.

This shift in prioritizing individual CAB interests to holistic cross‐CAB priorities can be seen in the card prioritization choices of the Shire Somali CAB, Faith Works CAB, and small group 1 (CAB leaders). The Shire Somali CAB discussed the need to better understand the prevalence and impact of autism within the Somali community and therefore categorized this project idea as most important during the first CAB‐specific card sorting activity. Similarly, the Faith Works CAB members highly prioritized projects aiming to better understand why people decline to participate in research. While the “work with primary care doctors to encourage more people to see a genetic counselor” card only reached mid‐importance levels during the CAB‐specific sorting activity, this topic was subsequently highly prioritized by the CAB leaders small group. Throughout the sequential card sorting activities, some topics rose in importance while others did not, which may reflect the transition from CAB‐specific priorities to projects addressing cross‐CAB goals through the use of small group discussions.

Ultimately, cross‐CAB members across the entire meeting came to the conclusion that a more universal, stepwise approach is needed to achieve community goals. CAB members expressed that selecting research projects that would generally improve access to and education about GC is needed *prior to* pursuing research projects that involve developing and implementing tailored GC approaches (such as GC counseling techniques, educational resources, etc.) for specific communities. As access to and education about GC services improves and more patients receive this care, cross‐CAB members noted that researchers could then turn their focus to developing a greater understanding of the nuanced needs of each community. Then, this knowledge should be applied to develop population‐specific strategies to improve community members' knowledge of, utilization of, and experience with GC services.

Lastly, cross‐CAB members collectively emphasized the critical need to utilize CBPR methodologies for all projects included in the final research agenda. Members shared that implementation of these research endeavors could bring up feelings of fear and mistrust of scientific/medical research, particularly related to genetics research, that many communities experience. Examples of fear highlighted by CAB members included concerns about potential misuse or abuse of genetic information, fear of being an “experiment,” and the belief that identifying a genetic risk will lead to disease manifestation. However, increased understanding of community beliefs could lead to greater cultural awareness, which cross‐CAB members noted is key to encouraging engagement in research, GC utilization, and positive health outcomes. Correspondingly, community‐based participatory projects present an opportunity to rebuild this trust and forge relationships between researchers and community partners to collaboratively pursue shared research goals.

## 4 Discussion

### 4.1 Summary

In this paper, we describe an innovative approach grounded in CBPR best practices that led to the creation of a prioritized GC research agenda with four distinct cultural CABs. The cultural identities, values, and prior experiences of each CAB differed, yet the top research topics spanned across cultures and communities to result in one collective voice urging GC researchers to pursue three topics: (A) working with primary care clinics to encourage more people to see a genetic counselor, (B) learning which GC techniques are best according to patients and the community, and (C) investigating whether educational projects make people more aware of GC or not. Given that all four CABs aligned their communities' needs, values, and goals around these three topics, it is profoundly evident that these resulting recommendations should be prioritized by researchers, funders, and governing bodies involved in guiding the future of GC research.

Qualitative card sorting methodologies and CBPR gold standard principles were combined in a unique and novel way in this study to facilitate the creation of a community‐driven prioritized GC research agenda. Card sorting activities have historically been used in psychology research but have also been effective in other settings, including community engagement studies in palliative care and resource allocation (Harrison and Taylor 2016; Meagher et al. 2024; Periyakoil et al. 2010). In the present study, card sorting activities were utilized to facilitate the development of distinctly ordered and prioritized lists of research topics that intrinsically centered CAB members' perspectives and viewpoints on the included topics (Donner 2001). Through intentional relationship‐building and co‐learning with the individual CABs as part of a longer‐term CBPR study (e.g., GC‐PRO study), the authors were able to develop a fundable research proposal with inherent community member support, expertise, and enthusiasm. When provided with the time and space, community‐researcher partnerships can lead to the identification of common themes across cultural populations that guide researchers to the most urgent and compelling community needs.

### 4.2 A Top Priority Topic: Increasing Access to GC Through Collaboration With Primary Care Clinics

Cross‐CAB members identified primary care clinics as a critical setting to promote GC awareness and uptake of GC services because primary and/or community care clinics are the most common source of medical care (Willis et al. 2020). In the United States, patients access specialty medical services including GC either through self‐referral or physician referral. Primary care providers therefore may be the first to recognize that a patient would benefit from a genetics evaluation and provide a referral to facilitate this (Pan et al. 2025). Thus, the primary care team (including physicians, nurses, clinic coordinators, community health workers, etc.) has a unique opportunity to identify and address patients' various needs, including those that relate to genetics.

The majority of research investigating barriers and strategies to integrating primary care and genetics services involve surveying medical personnel, such as primary care physicians, rather than the patient communities these services are designed to support (Carroll et al. 2021; Chou et al. 2021; Kenneson et al. 2025; Mikat‐Stevens et al. 2015; Seibel et al. 2022; Sharma et al. 2021; Srinivasan et al. 2021). Furthermore, interventions designed to increase utilization or awareness of genetics services within primary care settings are largely pre‐determined by research teams without any input from relevant community stakeholders (Bowler et al. 2025; Carroll et al. 2022; Kaphingst et al. 2021; Kerman et al. 2024; Scheuner et al. 2014). Through use of CBPR‐based approaches, community partners could help bridge the gap by advising clinicians and researchers on community‐ and patient‐driven interventions that could more effectively increase uptake and accessibility of genetics services in primary care settings.

Additionally, many research interventions are unfortunately short‐lived and/or struggle to contribute to sustainable solutions that positively impact overall community health (Altman 1995; Bogart and Uyeda 2009; Braithwaite et al. 2020; Hanney et al. 2015; Shelton et al. 2018). Employing a CBPR approach could empower researchers to build long‐term partnerships with key community leaders and ensure continued, bi‐directional feedback to support longevity of interventions aimed at improving genetics service utilization in primary care (Abu‐Omar et al. 2021; Bogart and Uyeda 2009; Wallerstein et al. 2020).

### 4.3 A Top Priority Topic: Aligning GC Techniques With Preferences of Patients and Communities

An abundance of studies have described patients' perspectives of and experiences with genetic counseling services within a variety of clinical settings and populations (Brown et al. 2021; Crook et al. 2022; Garza et al. 2020; Greve et al. 2020; Guimarães et al. 2013; Pichini et al. 2016; Semaka and Austin 2019; Skirton 2001). However, the vast majority of studies do not delineate which specific counseling techniques or communication strategies impacted the patient's experience, and in what ways. It is clear that patients report a variety of positive psychosocial and health outcomes as a result of GC sessions; however, the exact mechanisms and counseling techniques that lead to those patient experiences remain largely unelucidated. Most importantly, the effectiveness of various counseling techniques may differ by patients' racial/ethnic identities, cultural backgrounds, and other demographic descriptors (Dron et al. 2023; Joseph et al. 2019; Rolle et al. 2022; Shete et al. 2023); therefore, including underserved and racial/ethnic minoritized communities in studies of preferred GC communication strategies may help ensure findings are applicable to the diverse and multifaceted patient communities living in the United States. Just as these approaches may be advantageous in primary care, the integration of CBPR principles in studies investigating GC processes may also promote equitable, patient‐centered practices in GC, which are recommended by various healthcare governing bodies such as the National Academy of Medicine (Bau et al. 2019; Institute of Medicine (US) Committee on Quality of Health Care in America 2001).

### 4.4 A Top Priority Topic: Educational Initiatives to Investigate GC Awareness Within Communities

Education on and awareness of the benefits of genomic healthcare services is critical to ultimately improving community health, yet public awareness of GC remains low particularly in underserved communities (Canedo et al. 2019; Hann et al. 2017). A variety of CBPR‐based studies have focused on strategies to increase knowledge and awareness of genetics‐related health topics within underserved communities (Ali et al. 2019; Avey et al. 2016; Barlow‐Stewart et al. 2006; Blocker et al. 2020; Lumpkins et al. 2020; Sanghavi et al. 2019), with many other non‐CBPR studies focusing on genetics education to patient communities as well (Khan et al. 2016; Manswell Butty et al. 2012; San Miguel‐Majors et al. 2020). While the exact need for increased awareness varies across populations, cross‐CAB members affirmed that awareness of GC services remains low in their communities. Limited knowledge about genetics and its utility in medicine as well as differing beliefs around concepts like inheritance may lead individuals to consider GC services as unnecessary parts of their routine healthcare (Barlow‐Stewart et al. 2006; Lumpkins et al. 2020). CAB members discussed that many community members are unaware of the link between their family's health history and the individual's own medical risk and care plan. Members of the care team could play a critical role in providing education to clarify the importance of genetic factors in medicine (e.g., by explaining why family history questionnaires can inform a patient's care management plan), thus helping patients recognize the utility of GC services in managing their own health.

In addition to educational strategies at the patient or individual level, adopting these strategies with community input that are tailored at the organizational (e.g., community‐based organizations, CBOs), community (e.g., networks of CBOS), and societal levels (e.g., mass media) may enhance community awareness and other GC educational outcomes. Community engagement with the aim to address patient education thus becomes a process where community members are actively engaged and shaping the “ecology of genetic communication” (Maibach et al. 2007; Moran et al. 2016). This involves the community in identifying how to share a communication product (e.g., GC education) via highly utilized and trusted channels (Wilkin 2013).

### 4.5 Study Limitations

As a qualitative card sorting study with previously organized CAB members, this study has some limitations. First, this study utilized closed card sorting activities that included a limited number of project ideas. Hence, other research project ideas could have been generated independently by members of the cross‐CAB that were beyond the previous conversations but were not included in the current activities. However, the included project ideas were all previously generated by each CAB and had already been reviewed and approved by CAB members, thus allowing us to focus our time on ideas that had already been generated and vetted by multiple CAB participants at previous meetings. Further, the closed card sorting activities enabled efficient idea prioritization rather than day‐of idea generation in alignment with the goals of the cross‐CAB meeting, which was only a half‐day event. For these reasons, the research team chose to perform the content analysis to maximize efficiency but acknowledge that the cross‐CAB members were not involved in the analysis of CAB meeting content.

Second, participants were English‐speaking members from previously established CABs located primarily in the Midwest who had worked on prior GC projects. Other racial/ethnic minoritized communities (e.g., Native and Indigenous peoples, other Asian American communities, etc.) as well as representative populations from other geographical areas who are not English fluent and are not familiar with GC were not included in the study. As with all qualitative studies, the results are limited and cannot be generalized like quantitative results. Our results may also have been different if other members of the same communities who have different lived experiences and professional backgrounds had participated in the cross‐CAB. Nonetheless, they represent these community members' ideas and recommendations to improve GC and reduce genomic healthcare disparities.

## 5 Conclusions

Our cross‐CAB meeting resulted in prioritization of three top GC research areas, including: (A) work with primary care clinics to encourage more people to see a genetic counselor, (B) learn which GC techniques are best according to patients and the community, and (C) investigate whether educational projects make people more aware of GC or not. Our card sort methodologies and CBPR approach can serve as a model for other researchers to identify critical research directions and collaborate with relevant stakeholder groups in order to empower community voices to shape the research that best addresses their needs and to guide researchers on the next directions for their work. It is our hope that these community‐recommended research directions will be adopted by GC researchers and prioritized by funding bodies, thereby guiding future initiatives toward issues that are critical to these communities who are impacted by genomic healthcare disparities.

## Author Contributions

**Victoria R. Johnson:** conceptualization, methodology, data curation, formal analysis, writing – original draft, writing – review and editing. **Elena R. Fisher:** conceptualization, funding acquisition, data curation, writing – original draft, writing – review and editing. **Kathleen A. Culhane‐Pera:** conceptualization, data curation, writing – review and editing. **Crystal Y. Lumpkins:** conceptualization, data curation, writing – review and editing. **Abdirashid Shire:** conceptualization, data curation, writing – review and editing. **Mariana Ramírez:** conceptualization, data curation, writing – review and editing. **Aida Parra de Young:** data curation, writing – review and editing. **Carolina E. Uribe Abad:** data curation, writing – review and editing. **Eric Yang:** data curation, writing – review and editing. **Sey Lee:** data curation, writing – review and editing. **Abdiziz Abdi:** data curation, writing – review and editing. **J. Michelle Vann:** data curation, writing – review and editing. **Stephanie Moss:** data curation, writing – review and editing. **Heather A. Zierhut:** conceptualization, funding acquisition, methodology, data curation, formal analysis, writing – review and editing.

## Funding

This work was supported by the University of Minnesota Office of Discovery and Translation, and National Human Genome Research Institute (R01HG011916).

## Disclosure

AI statement: No artificial intelligence tools were used to develop this manuscript.

## Ethics Statement

This study was determined to be exempt from human subjects research regulations by the University of Minnesota Institutional Review Board. However, the authors followed standard ethical research principles, including providing information regarding informed consent to participate in the meeting to all individuals involved.

## Conflicts of Interest

The authors declare no conflicts of interest.

## Supporting information


**Table S1:** CAB‐specific project idea cards generated via content analysis. CAB‐specific sets of project idea cards are shown prior to subsequent categorization and prioritization. Cards are divided by general research topic area to highlight overlap and distinction across CABs.

## Data Availability

Data generated from the cross‐CAB meeting (including the results of each card sorting activity) are fully disclosed and described in this paper. Additional data that supports the findings of this study (e.g., facilitator notes, recordings, etc.) may be available on request from the corresponding author. The data are not publicly available due to privacy or ethical restrictions, and would require approval to share from the cross‐CAB members.
