# Supplementary material for: Community‐Driven Research Priorities for Genetic Counseling: Shaping the Future of Genetic Counseling Research by Centering Multicultural Perspectives
Source: J Genet Couns. 2026 Jul 28;35(4):e70270. doi: 10.1002/jgc4.70270 (PMC13411192; doi:10.1002/jgc4.70270)
Supplement: Supplementary file 1 — Table S1: CAB‐specific project idea cards generated via content analysis. CAB‐specific sets of project idea cards are shown prior to subsequent categorization and prioritization. Cards are divided by general research topic area to highlight overlap and distinction across CABs. [file JGC4-35-0-s001.pdf]

CAB-Specific Project Idea Cards Generated via Content Analysis				
**Topic area**	**Faith Works CAB**	**JUNTOS CAB**	**Shire Somali CAB**	**Hmong CAB**
**Work with primary care clinics**	Work with primary care doctors to encourage more people to see a genetic counselor.		Work with primary care doctors to encourage more people to see a genetic counselor.	
				Learn about how clinics add new educational materials to their current working systems.
**Genetic counseling techniques**	Learn which genetic counseling techniques are best according to patients.	Learn which genetic counseling techniques are best according to patients.	Learn which genetic counseling techniques are best according to patients.	
		Compare the impact of genetic counseling (or other medical visits) among members of different communities.		
**Community education**	Create and carry out educational projects for the community.	Create and carry out educational projects for the community.		
			Investigate whether educational projects make people more aware of genetic counseling or not.	Investigate whether educational projects make people more aware of genetic counseling or not.
**Challenges to conducting research**	Understand why people say “no” when asked to be part of research projects.			Understand why people say “no” when asked to be part of research projects.
		Translate patient surveys to Spanish and/or other languages so that they can be used for genetic counseling research.		
**Epidemiology**			Learn about genetic medical conditions that are more common in certain communities (for example, autism).	
**Demographics**	Compare demographics of people who see genetic counselors to demographics of local communities.	Compare demographics of people who see genetic counselors to demographics of local communities.	Compare demographics of people who see genetic counselors to demographics of local communities.	Compare demographics of people who see genetic counselors to demographics of local communities.
				Ask how people like to identify their own demographic information (such as religion, race, ethnicity, clan name, language, among others).
